# Risk of Postpartum Depression and Postpartum Psychosis in Patients With Obstetric Complications: A Case-Control Study

**DOI:** 10.7759/cureus.86156

**Published:** 2025-06-16

**Authors:** Muskan Z Kamal, Nida Naseer, Abdar A Khan, Zarar A Khan, Muhammad Azeem Hayat, Yasir Mahmood, Tabinda Danish

**Affiliations:** 1 Department of Community Medicine, Khyber Medical College, Peshawar, Peshawar, PAK; 2 Department of Internal Medicine, Khyber Teaching Hospital, Peshawar, PAK; 3 Department of Pharmacology, Peshawar Medical College, Peshawar, PAK; 4 Department of Surgery, Khyber Teaching Hospital, Peshawar, PAK; 5 Department of Medicine, Khyber Teaching Hospital, Peshawar, PAK

**Keywords:** hemorrhage, hypertensive disorders, mental health, obstetric complication, postpartum depression, preterm birth, risks

## Abstract

Background: Postpartum psychiatric disorders, including postpartum depression (PPD) and postpartum psychosis (PPP), are major contributors to maternal morbidity and adverse child outcomes globally. While psychosocial risk factors are well established, the potential mechanistic role of obstetric complications in precipitating these conditions remains less clearly delineated.

Objective: This study aimed to evaluate the association between specific obstetric complications and the risk of postpartum depression and psychosis within six months after delivery, using a biopsychosocial framework.

Methods: We conducted a retrospective, matched case-control study using medical records from Khyber Teaching Hospital, Peshawar (Pakistan), covering deliveries from January 2018 to December 2023. Cases included women aged 18-45 years diagnosed with PPD or PPP within six months postpartum based on DSM-5 criteria. Controls were women who delivered during the same period without psychiatric diagnoses, matched 2:1 by age, parity, and delivery mode. Obstetric complications assessed included preeclampsia/eclampsia, gestational diabetes mellitus (GDM), postpartum hemorrhage, preterm birth, placental abruption, intrauterine growth restriction (IUGR), chorioamnionitis, and cesarean delivery. Conditional logistic regression was used to calculate adjusted odds ratios (aOR) with 95% confidence intervals (CI).

Results: A total of 220 cases and 440 matched controls were analyzed. Preeclampsia/eclampsia (aOR: 2.30; 95% CI: 1.45-3.64; p<0.001), postpartum hemorrhage (aOR: 2.10; 95% CI: 1.30-3.38; p=0.002), preterm birth (aOR: 1.85; 95% CI: 1.20-2.85; p=0.004), and GDM (aOR: 1.55; 95% CI: 1.00-2.40; p=0.049) were significantly associated with increased risk of postpartum psychiatric disorders. No significant associations were found for placental abruption, IUGR, chorioamnionitis, or cesarean delivery.

Conclusion: Obstetric complications-particularly preeclampsia/eclampsia, postpartum hemorrhage, preterm birth, and gestational diabetes-may act as physiological stressors that interact with underlying vulnerabilities, consistent with stress-diathesis and inflammatory models of psychopathology. These findings support a multifactorial conceptualization of postpartum psychiatric illness and emphasize the importance of integrating obstetric risk profiles into postpartum mental health screenings. In low-resource settings like Pakistan, targeting women with complicated deliveries and known psychosocial risk factors through collaborative, interdisciplinary care models can help prevent chronic maternal mental health conditions and improve developmental outcomes for children.

## Introduction

Postpartum psychiatric disorders represent a major public health concern, affecting not only the health and well-being of mothers but also the development and safety of their children. Postpartum depression (PPD) affects approximately 10-20% of women globally, while postpartum psychosis (PPP), although rarer, carries substantial morbidity and potential risk for both maternal and infant mortality if left untreated [1]. The first six months after childbirth are recognized as a critical window during which psychiatric symptoms often emerge, yet the underlying risk factors that predispose women to these disorders remain incompletely understood.

While psychological and social factors such as prior history of depression, lack of social support, and socioeconomic hardship have been consistently implicated in the etiology of postpartum psychiatric illnesses [2], increasing attention has turned toward biological and obstetric contributors. Emerging evidence suggests that complications arising during pregnancy and delivery, such as hypertensive disorders, gestational diabetes mellitus (GDM), postpartum hemorrhage (PPH), preterm birth, and infections, may contribute to the pathophysiology of postpartum psychiatric outcomes through inflammatory, neuroendocrine, and stress-related mechanisms [3,4].

Studies examining these associations, however, have produced mixed results. Some investigations have reported significant links between specific obstetric complications and the onset of PPD [5,6], while others have found no independent associations after accounting for psychological or sociodemographic factors. For example, Gausia et al. found that after adjusting for psychosocial variables, obstetric complications did not significantly predict PPD [7]. Similarly, Leigh and Milgrom reported that psychosocial risk factors such as prior mental health history and low social support were stronger predictors of postnatal depression than obstetric events [8]. Josefsson et al. also demonstrated that obstetric and somatic complications were not independently associated with PPD when psychological history was considered [9]. Furthermore, much of the existing literature focuses predominantly on PPD, with relatively limited exploration into more severe outcomes such as PPP. Sit et al. emphasized the relative paucity of research on PPP despite its clinical severity [10], and Bergink et al. similarly noted the underrepresentation of PPP in perinatal mental health studies [11]. Meltzer-Brody and Stuebe further highlighted that although PPP carries a high burden of morbidity and mortality, it remains significantly understudied, despite potential long-term psychiatric and cardiometabolic consequences [12]. Given the profound clinical implications of these disorders, clarifying the role of obstetric complications is vital for improving screening, prevention, and early intervention strategies.

Therefore, the aim of this study was to investigate the association between obstetric complications and the risk of developing PPD and PPP within six months after delivery, using a matched case-control design. We hypothesized that women who experienced specific obstetric complications, including hypertensive disorders, GDM, PPH, preterm birth, and infections, would have a significantly higher risk of postpartum psychiatric disorders compared to women without such complications, independent of pre-existing psychological and socioeconomic factors.

## Materials and methods

Study design and setting

This retrospective case-control study was conducted at Khyber Teaching Hospital, Peshawar, Pakistan, a major tertiary care center with approximately 12,000 deliveries annually [13]. The study utilized medical records spanning deliveries from January 2018 to December 2023. The research followed the Strengthening the Reporting of Observational Studies in Epidemiology (STROBE) guidelines [14] to ensure methodological rigor.

Ethical considerations

The study protocol was approved by the Institutional Review Board of Khyber Teaching Hospital (approval number: 967/DM/KMC). To maintain patient confidentiality, all data were de-identified during extraction and analysis. The study procedures conformed to the ethical principles outlined in the Declaration of Helsinki [15].

Inclusion and exclusion criteria

Women aged between 18 and 45 years, who had delivered a live-born infant and had complete obstetric and psychiatric records available, were considered eligible for inclusion. Women were excluded if they had a history of severe pre-pregnancy psychiatric disorders such as schizophrenia or bipolar disorder, if they had experienced a stillbirth or neonatal death within 24 hours postpartum, or if their medical records were incomplete.

Participant selection

Cases 

Cases were defined as women aged 18-45 years who delivered a live infant and received a confirmed clinical diagnosis of PPD or PPP within six months of delivery. Although the Diagnostic and Statistical Manual of Mental Disorders, Fifth Edition (DSM-5) specifies a peripartum onset of within four weeks [16], and the International Classification of Diseases, Tenth Revision (ICD-10) uses a six-week threshold [17], we adopted a broader six-month timeframe. This decision reflects emerging clinical and epidemiological consensus acknowledging delayed-onset postpartum psychiatric presentations, particularly in low-resource settings where follow-up may be delayed. All diagnoses were confirmed by trained psychiatrists based on DSM-5 criteria, with detailed clinical history ensuring consistency regarding symptom onset.

Controls

Controls were women who delivered during the same time frame and shared similar obstetric characteristics but had no documented psychiatric diagnosis within six months postpartum. Each case was matched with two controls based on age (±2 years), parity, and mode of delivery. Medical records of control participants were reviewed over the same six-month postpartum period to confirm the absence of psychiatric symptoms.

Exposure assessment

The primary exposures of interest were obstetric complications occurring during pregnancy, labor, or delivery. These included hypertensive disorders of pregnancy (e.g., preeclampsia and eclampsia), GDM, PPH, preterm birth (defined as delivery before 37 weeks of gestation), placental abruption, antepartum hemorrhage due to placenta previa, intrauterine growth restriction (IUGR), chorioamnionitis and postpartum infections, and cesarean delivery. The presence of these complications was identified through documented clinical diagnoses and confirmed using corresponding ICD-10 codes.

Psychiatric assessment and DSM-5 criteria

Initial mental health screening was performed using the Edinburgh Postnatal Depression Scale (EPDS) [18,19], administered during routine postpartum visits. Women scoring ≥13 were referred for comprehensive psychiatric evaluation. Final diagnoses of PPD or PPP were confirmed by licensed psychiatrists using DSM-5 diagnostic criteria.

Data collection

Data were collected by two trained researchers (MZK and AAK) using a standardized electronic extraction form (see Appendices). This included maternal sociodemographic and clinical characteristics, obstetric history, and psychiatric information. The psychiatric history was recorded in two categories. Exclusionary psychiatric diagnoses, such as schizophrenia or bipolar disorder, led to case exclusion. Non-exclusionary psychiatric history, such as antenatal depression, anxiety disorders, adjustment disorders, or prior use of psychiatric medications, which were included as covariates in regression models. All psychiatric information was gathered from comprehensive clinical notes, antenatal visit summaries, and mental health referrals documented in the medical record. Information on infant feeding practices during hospital stay, whether exclusive breastfeeding, formula feeding, or mixed feeding, was also recorded when documented. In case of discrepancies, a third reviewer (NN) was consulted for consensus.

Sample size calculation

Sample size estimation was based on an expected odds ratio (OR) of 2.0, 80% power, and a 5% significance level [20]. Assuming 25% exposure among controls and a 2:1 control-to-case ratio, the minimum required sample size was 156 cases and 312 controls. To account for data loss and allow for confounder adjustment, we inflated the sample size by 25%. The final study included 220 cases and 440 matched controls.

Statistical analysis

All statistical analyses were conducted using IBM SPSS Statistics for Windows, version 23 (Released 2015; IBM Corp., Armonk, New York, United States). Continuous variables were assessed for normality and summarized using mean and standard deviation (SD) for normally distributed data, or medians and interquartile ranges for skewed data. Independent t-tests were used to compare continuous variables between groups. Categorical variables were presented as frequencies and percentages, and compared using the Chi-square (χ²) test. To evaluate associations between obstetric complications and the risk of PPD or PPP, conditional logistic regression was used. Results are reported as adjusted OR (aOR) with 95% confidence interval (CI). The Z-test was applied to test the significance of regression coefficients in the logistic models. Multivariable logistic regression models were constructed to adjust for relevant confounders, including maternal education, income status, pre-pregnancy mental health history, substance use during pregnancy, and level of social support. Multicollinearity was assessed using variance inflation factors (VIF), and a significance level of p < 0.05 was used (p < 0.001 considered highly significant).

Handling of missing data

Missing data were managed using multiple imputation with chained equations (MICE), under the assumption that the data were missing at random. Ten imputed datasets were created, and final estimates were combined using Rubin’s rules to produce unbiased parameter estimates and standard errors [21].

Sensitivity analyses

To assess the robustness of the findings, sensitivity analyses were conducted by restricting the study sample to primiparous women and by excluding women with any history of antenatal depression or anxiety. Stratified analyses based on mode of delivery (cesarean versus vaginal delivery) were performed to evaluate whether delivery method influenced the association between obstetric complications and postpartum psychiatric outcomes.

## Results

Among the 220 women diagnosed with PPD or PPP, the mean age was 30.5 ± 4.2 years compared to 30.2 ± 4.3 years in the 440 controls, with no statistically significant difference (t = 0.75, p = 0.45). Most participants in both groups were married, with 205 (93.2%) cases and 418 (95.0%) controls reporting marriage; the distribution across marital status subcategories, widowed (six (2.7%) cases vs. nine (2.0%) controls), divorced (three (1.4%) vs. six (1.4%)), and undocumented (six (2.7%) vs. seven (1.6%)), did not significantly differ (χ² = 1.12, p = 0.29). Women with postpartum psychiatric disorders were significantly more likely to have low socioeconomic status, with 99 (45.0%) cases versus 132 (30.0%) controls (χ² = 11.16, p = 0.001), and a history of mental illness, reported in 55 (25.0%) cases versus 35 (8.0%) controls (χ² = 31.14, p < 0.001). Substance use during pregnancy was also more prevalent among cases, observed in 40 (18.2%) compared to 40 (9.1%) controls (χ² = 9.04, p = 0.003). While a lower proportion of cases had adequate antenatal visits: 171 (77.7%) vs. 369 (83.9%); this difference did not reach statistical significance (χ² = 3.00, p = 0.08). These findings, summarized in Table 1, underscore the importance of socioeconomic disadvantage, prior psychiatric history, and prenatal substance use as key non-obstetric risk factors for postpartum psychiatric disorders.

**Table 1:** Baseline characteristics of cases and controls ^†^ Independent t-test was used; ^§^ Chi-square (χ²) test was used; *Significant at p < 0.05; ^**^Highly significant at p < 0.001. All variables listed were assessed as potential confounders and included in multivariable conditional logistic regression models. Matching was performed on age, parity, and delivery mode. Variables were selected based on clinical relevance and prior evidence.

Variable	Subcategory	Cases (n=220)	Controls (n=440)	Test Statistic	p-value
Age (years)	Mean ± SD	30.5 ± 4.2	30.2 ± 4.3	0.75 ^†^	0.45
Marital Status	Married	205 (93.2%)	418 (95.0%)	1.12 ^§^	0.29
	Widowed	6 (2.7%)	9 (2.0%)		
	Divorced	3 (1.4%)	6 (1.4%)		
	Undocumented	6 (2.7%)	7 (1.6%)		
Socioeconomic Status	Low	99 (45.0%)	132 (30.0%)	11.16 ^§^	0.001*
	Middle/High	121 (55.0%)	308 (70.0%)		
History of Mental Illness	Present	55 (25.0%)	35 (8.0%)	31.14 ^§^	<0.001**
	Absent	165 (75.0%)	405 (92.0%)		
Substance Use in Pregnancy	Yes	40 (18.2%)	40 (9.1%)	9.04 ^§^	0.003*
	No	180 (81.8%)	400 (90.9%)		
Antenatal Visits	Adequate (≥4 visits)	171 (77.7%)	369 (83.9%)	3.00 ^§^	0.08
	Inadequate (<4 visits)	49 (22.3%)	71 (16.1%)		

Obstetric complications were significantly more prevalent among women who developed PPD or PPP. Preeclampsia or eclampsia occurred in 48 (22%) cases versus 44 (10%) controls (χ² = 15.60, p < 0.001), and GDM was reported in 18% of cases compared to 12% of controls (χ² = 4.82, p = 0.03). PPH was more frequent in cases (n=35; 16%) than controls (n=35;8%) (χ² = 9.44, p = 0.002). Similarly, preterm birth was notably higher in cases (n=53; 24%) compared to controls (n=62; 14%) (χ² = 10.53, p = 0.001). Differences in rates of placental abruption, IUGR, chorioamnionitis, and cesarean delivery were observed but did not reach statistical significance (all p > 0.05). These results, as shown in Table 2, imply that specific obstetric complications, especially hypertensive disorders, hemorrhage, and preterm birth, may contribute significantly to the postpartum psychiatric burden.

**Table 2:** Obstetric complications among cases and controls Statistical test used: Chi-square (χ²) test for all comparisons; ^*^Significant p-value < 0.05; ^**^Highly significant p-value < 0.001.

Obstetric Complication	Cases (n=220), n (%)	Controls (n=440), n (%)	χ² value	p-value
Preeclampsia/Eclampsia	48 (22%)	44 (10%)	15.60	<0.001**
Gestational Diabetes Mellitus	40 (18%)	53 (12%)	4.82	0.03**
Postpartum Hemorrhage	35 (16%)	35 (8%)	9.44	0.002**
Preterm Birth (<37 weeks)	53 (24%)	62 (14%)	10.53	0.001**
Placental Abruption	11 (5%)	9 (2%)	3.02	0.08
Intrauterine Growth Restriction	22 (10%)	26 (6%)	2.53	0.12
Chorioamnionitis	13 (6%)	13 (3%)	2.08	0.15
Cesarean Delivery	88 (40%)	154 (35%)	1.31	0.25

After adjusting for potential confounders, several obstetric complications remained significantly associated with an elevated risk of postpartum psychiatric disorders. Women with a history of preeclampsia or eclampsia had an aOR of 2.30 (95%CI: 1.45-3.64, Z = 3.45, p < 0.001). Those with GDM had a 1.55-fold increased risk (95%CI: 1.00-2.40, Z = 1.96, p = 0.049), while PPH was associated with a 2.10-fold higher risk (95%CI: 1.30-3.38, Z = 3.06, p = 0.002). Preterm birth was also independently linked to postpartum psychiatric disorders with an aOR of 1.85 (95%CI: 1.20-2.85, Z = 2.88, p = 0.004). Associations with placental abruption, IUGR, chorioamnionitis, and cesarean delivery were not statistically significant after adjustment. These findings, as shown in Table 3, confirm that certain obstetric complications, even after controlling for other factors, act as independent predictors of postpartum psychiatric morbidity.

**Table 3:** Association between obstetric complications and risk of PPD/PPP: adjusted conditional logistic regression Statistical test used: Z-test for evaluating regression coefficients; *Significant p-value < 0.05; **Highly significant p-value < 0.001. PPD: postpartum depression; PPP: postpartum psychosis

Obstetric Complication	Adjusted Odds Ratio	95% Confidence Interval	Z value	p-value
Preeclampsia/Eclampsia	2.3	1.45 – 3.64	3.45	<0.001**
Gestational Diabetes Mellitus	1.55	1.00 – 2.40	1.96	0.049*
Postpartum Hemorrhage	2.1	1.30 – 3.38	3.06	0.002*
Preterm Birth	1.85	1.20 – 2.85	2.88	0.004*
Placental Abruption	1.9	0.80 – 4.50	1.51	0.13
Intrauterine Growth Restriction	1.4	0.75 – 2.60	1.08	0.28
Chorioamnionitis	1.7	0.80 – 3.70	1.44	0.15
Cesarean Delivery	1.2	0.85 – 1.70	1.08	0.28

**Figure 1:**
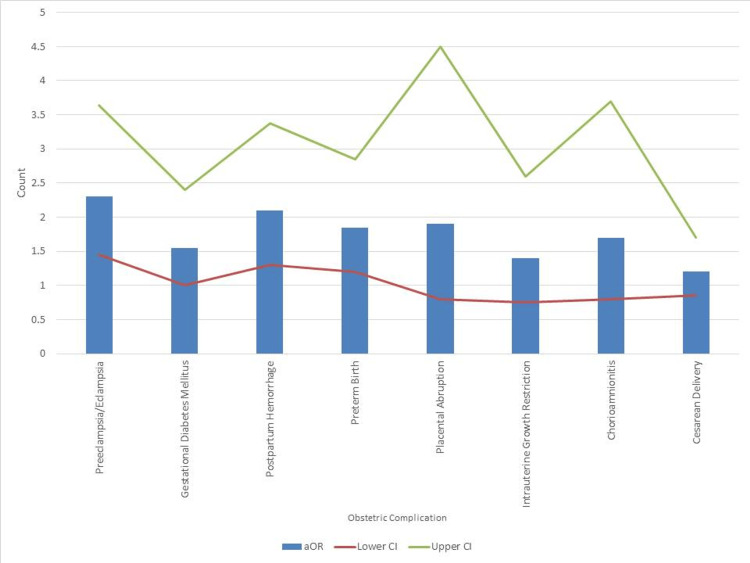
Adjusted odds ratios (aORs) for obstetric complications associated with postpartum psychiatric disorders

Figure 1 is based on aORs highlighted preeclampsia/eclampsia (aOR = 2.30, 95%CI: 1.45-3.64), PPH (aOR = 2.10, 95%CI: 1.30-3.38), preterm birth (aOR = 1.85, 95% CI: 1.20-2.85), and GDM (aOR = 1.55, 95%CI: 1.00-2.40) as statistically significant risk factors for PPD or PPP. Obstetric conditions such as placental abruption (aOR = 1.90), IUGR (aOR = 1.40), chorioamnionitis (aOR = 1.70), and cesarean delivery (aOR = 1.20) showed elevated odds but did not achieve statistical significance. The summarized effect estimates suggest that hypertensive disorders, hemorrhagic events, and premature delivery are the most clinically impactful obstetric predictors for postpartum mental health outcomes.

## Discussion

In this case-control study, we observed that women who developed PPD or PPP were significantly more likely to have experienced specific obstetric complications, particularly preeclampsia, GDM, PPH, and preterm birth. These associations persisted even after adjusting for sociodemographic and psychiatric confounders, suggesting that certain pregnancy-related morbidities may play an independent role in the pathogenesis of postpartum psychiatric disorders.

The strong association between hypertensive disorders of pregnancy and postpartum psychiatric outcomes aligns with previous research. Meltzer-Brody et al. demonstrated that preeclampsia nearly doubled the risk of postpartum psychiatric illness across a large multinational cohort [6]. Similarly, Caropreso et al. performed a meta-analysis confirming preeclampsia's role as a significant predictor of PPD and PPP, potentially due to overlapping vascular and neuroinflammatory mechanisms [22].

The observed association between GDM and PPD (aOR = 1.55) is consistent with earlier findings. Hinkle et al. reported that women with GDM had 1.4 times higher odds of experiencing depressive symptoms at six weeks postpartum [23]. This link may be explained by the systemic inflammation, glycemic dysregulation, and hormonal changes associated with GDM. Runkle et al. further supported this in a large United States birth cohort, revealing bidirectional associations between perinatal mood disorders and GDM [24].

PPH was another significant factor, with affected women facing a two-fold increase in risk of PPD/PPP. Deniau et al., in a national French cohort, found a similar pattern, highlighting the psychological trauma and physical stress of hemorrhagic events as key contributors to maternal psychiatric vulnerability [25].

Preterm birth was also independently associated with postpartum psychiatric disorders in our study (aOR = 1.85). Vigod et al. found that mothers of preterm infants were 1.5 times more likely to develop PPD [26]. Alturki et al. found that while there was no correlation between mode of delivery and PPD, emergency cesarean section could contribute to PPD [27]. The stress of neonatal ICU care, disrupted bonding, and unexpected early delivery are all potential mechanisms contributing to this risk.

Placental abruption, IUGR, and chorioamnionitis showed elevated but non-significant odds ratios. This is aligned with a study by Kuklina et al., which found weaker and inconsistent associations for these conditions with postpartum mental health disorders [28]. Similarly, cesarean delivery was not significantly associated with PPD/PPP after adjustment, echoing conclusions from a systematic review by Xu et al., which found no consistent link between mode of delivery and mental health outcomes [29].

Importantly, our findings affirm that obstetric events alone do not fully account for postpartum psychiatric risk. Psychosocial and clinical variables, such as prior psychiatric illness, substance use during pregnancy, and low socioeconomic status, were also significant predictors, consistent with the biopsychosocial model of perinatal mental health. Kang-Yi et al. emphasized this complexity in a population-based study, urging integrated mental health care alongside obstetric follow-up [30].

Strengths and limitations

This study has several notable strengths. It utilized a large, well-defined sample from a major tertiary care hospital over a six-year period. Rigorous matching of controls by age, parity, and delivery mode helped reduce selection bias. The use of DSM-5 diagnostic criteria, confirmed through psychiatric evaluation by licensed professionals, ensured diagnostic reliability, though we acknowledge that diagnostic consistency may still vary in retrospective data. Comprehensive clinical records allowed for accurate classification of prior psychiatric history, enabling effective confounder adjustment in multivariable models. The study followed STROBE guidelines and employed robust statistical methods, including adjusted conditional logistic regression with effect size reporting and sensitivity analyses, thereby strengthening the validity of the conclusions.

However, some limitations should be acknowledged. The retrospective design poses inherent risks of information and selection bias, although these were minimized through standardized data abstraction and independent validation by trained reviewers. While efforts were made to collect information on variables such as infant feeding practices, the data were inconsistently documented and could not be included in the analysis. Additionally, the single-center design may limit generalizability to other populations, particularly rural or private healthcare settings. The relatively small number of placenta previa cases may have affected the precision of related estimates. Finally, we recognize the value of including a third comparison group of women with obstetric complications but without psychiatric disorders to identify potential protective factors; however, this was not feasible given the absence of routine postpartum psychiatric screening in the medical records. This limitation has been highlighted as a key area for future prospective research.

Future Directions

Future research should focus on prospective, multicenter studies to improve generalizability and establish causal relationships between obstetric complications and postpartum psychiatric disorders. Including a third comparison group, women who experienced obstetric complications but did not develop PPD or PPP, could provide helpful information regarding protective psychosocial, clinical, or relational factors. Standardized data collection tools, including validated mental health screening questionnaires and structured antenatal/postnatal assessments conducted by trained personnel, will be essential.

Incorporating biological markers, genetic susceptibility data, and social determinants of health will help elucidate underlying mechanisms and contextual risks. Routine postpartum psychiatric screening, especially for women with complications such as preeclampsia, GDM, hemorrhage, or preterm birth, should be integrated into follow-up care to support early identification and intervention. Furthermore, the development of context-specific risk prediction models and scalable mental health strategies embedded within maternal health services is crucial for improving outcomes in low- and middle-income countries. Studies should also explore the role of stigma, healthcare access, and family dynamics in shaping help-seeking behaviors to inform more responsive and inclusive care frameworks.

## Conclusions

In this case-control study, we found that specific obstetric complications, particularly preeclampsia, GDM, PPH, and preterm birth-were independently associated with a significantly increased risk of PPD and PPP within six months after delivery. These associations remained robust after adjusting for sociodemographic and psychiatric confounders, underscoring the biopsychosocial nature of postpartum psychiatric morbidity. The findings align with stress-diathesis and inflammatory models of mental illness, suggesting that obstetric stressors may act as physiological or psychosocial triggers in biologically or psychologically vulnerable individuals. Furthermore, non-obstetric risk factors such as low socioeconomic status, prior mental health history, and substance use during pregnancy reinforce the multifactorial etiology of postpartum disorders. These results support the need for integrated obstetric and mental health screening strategies rooted in a systems-based approach to maternal care.

## Appendices

**Table 4:** Structured Data Collection Questionnaire PPD: postpartum depression; PPP: postpartum psychosis; DSM-5: Diagnostic and Statistical Manual of Mental Disorders, Fifth Edition; NICU: neonatal intensive care unit

Section	Variable	Response Options / Format
Sociodemographic Data	Maternal Age	_____ (Years)
	Ethnicity	Pashtun / Punjabi / Baloch / Sindhi / Other
	Marital Status	Married / Divorced / Widowed / Undocumented
	Socioeconomic Status	Low / Middle / High (Based on income & occupation)
	Education Level	Illiterate / Primary / Secondary / Higher
	Employment Status	Employed / Unemployed / Informal sector
	Urban/Rural Residence	Urban / Rural
	Household Size	_______
	Access to Health Facility	Yes / No
Obstetric History	Parity	_______
	Gravidity	_______
	Mode of Delivery	Vaginal / Cesarean / Assisted
	Antenatal Care Visits	Adequate (>=4) / Inadequate (<4)
	Hypertensive Disorders	Yes / No
	Gestational Diabetes Mellitus	Yes / No
	Postpartum Hemorrhage (PPH)	Yes / No
	Preterm Birth (<37 weeks)	Yes / No
	Placental Abruption	Yes / No
	Placenta Previa/Antepartum Hemorrhage	Yes / No
	Intrauterine Growth Restriction	Yes / No
	Chorioamnionitis	Yes / No
	Postpartum Infection	Yes / No; Specify if available _______
Mental Health History	Personal Psychiatric History	Yes / No; If Yes: Diagnosis, Treatment, Duration
	Family Psychiatric History	Yes / No; If Yes: Relation, Diagnosis
	Substance Use During Pregnancy	Yes / No; If Yes: _______ (e.g., tobacco, drugs)
	Past Treatment for Depression/Anxiety	Yes / No
	EPDS Score	Numeric (0–30); Cutoff ≥13
	Final Psychiatric Diagnosis	PPD / PPP / None (DSM-5 confirmed)
Postpartum/Infant Factors	Feeding Practice	Exclusive Breastfeeding / Formula / Mixed
	Breastfeeding Initiation (Time)	Within 1 hour / >1 hour / Not Initiated
	NICU Admission	Yes / No; If Yes: Reason _______
	Infant Outcome at Discharge	Alive / Deceased
	Infant Gender	Male / Female
	Birth Weight	Numeric (kg)
Social Support	Support During Pregnancy	Yes / No; If Yes: _______ (Spouse, Family, NGO)
	Postpartum Support	Yes / No; If Yes: _______
	Domestic Violence Exposure	Yes / No / Not Documented
	Perceived Stress Level	Low / Moderate / High
